# Community and Systems Contributors and Strategies to Reduce Racial Inequities in Maternal Health in the Deep South: Provider Perspectives

**DOI:** 10.1089/heq.2023.0114

**Published:** 2023-09-13

**Authors:** Molly B. Richardson, Angelina A. Toluhi, Monica L. Baskin, Henna Budhwani, Zoë I. Julian, Candace C. Knight, Rachel Sinkey, Jeff M. Szychowski, Alan T.N. Tita, Martha S. Wingate, Janet M. Turan

**Affiliations:** ^1^ Department of Health Policy and Organization, University of Alabama at Birmingham (UAB), Birmingham, Alabama, USA.; ^2^ Department of Medicine, University of Alabama at Birmingham (UAB), Birmingham, Alabama, USA.; ^3^ Center for Women's Reproductive Health, UAB, Birmingham, Alabama, USA.; ^4^ Center of Population Sciences for Health Equity, College of Nursing, Florida State University (FSU), Tallahassee, Florida, USA.; ^5^ Department of Obstetrics and Gynecology, WellStar Kennestone Regional Medical Center, Marietta, Georgia, USA.; ^6^ School of Nursing, UAB, Birmingham, Alabama, USA.; ^7^ Department of Obstetrics and Gynecology, UAB, Birmingham, Alabama, USA.; ^8^ Department of Biostatistics, UAB, Birmingham, Alabama, USA.

**Keywords:** equity, maternal health, community, systems, disparities, racism

## Abstract

**Purpose:**

Black pregnant individuals in Alabama are disproportionately affected by severe maternal morbidity and mortality (SMM). To understand why racial disparities in maternal health outcomes persist and identify potential strategies to reduce these inequities, we sought perspectives from obstetric health care providers, health administrators, and members of local organizations who provide pregnancy, delivery, and postpartum care services in Alabama.

**Methods:**

We conducted qualitative in-depth interviews with stakeholders (*n*=20), purposively recruited from community-based organizations, clinical settings, government organizations, and academic institutions. Interview guides were based on Howell's conceptual model of pathways to racial disparities in maternal mortality. Data were coded using a modified framework theory approach and analyzed thematically.

**Results:**

Racism, unjust laws and policies, and poverty/lack of infrastructure in communities emerged as major themes contributing to racial disparities in maternal health at the community and systems levels. Inadequate health insurance coverage was described as a strong driver of the disparities. Service providers suggested strategies for Alabama should be community focused, evidence based, and culturally sensitive. These should include Medicaid expansion, expanded parental leave, and removal of laws restricting choice. Community- and systems-level interventions should include community infrastructure improvements, choice in maternity services, and provision of digital communication options.

**Conclusions:**

Providers shared perspectives on community and structural areas of intervention to reduce racial inequities in SMM. These results can inform discussions with health system and community partners about Alabama and other Deep South initiatives to improve maternal health outcomes in black communities.

## Introduction

Black pregnant individuals in the United States are disproportionately affected by severe maternal morbidity and mortality (SMM).^1–4^ Alabama has the third worst maternal mortality ratio in the United States, with similar differences by race/ethnicity.^5^ The causes for these disparities are complex and incompletely understood, with multiple contributing factors at the individual, community, provider, health care organization, and system levels.^6–8^ To understand the drivers of disparities, it is essential to acknowledge race as a social construct and examine the many specific barriers to health care utilization for pregnant individuals driven by social and economic inequities rooted in historical and ongoing racism.^9–11^ There are calls to understand better the effects of income inequality and structural racism on health at a state level and noted research gaps (e.g., engagement and inclusion of community perspectives and social determinants).^12,13^

Ongoing efforts at state and local levels to improve maternal health in Alabama include the establishment of a maternal mortality review committee, the Alabama Perinatal Quality Collaborative, and the initiation of state reporting on maternal death data by race/ethnicity.^5,14^ Racial disparities persist despite current evidence and ongoing efforts to reduce this inequity.^15^

We used a framework approach to conduct and analyze in-depth interviews to identify community and systems factors contributing to these disparities in Alabama and, importantly, strategies to overcome them.^16^ We sought the perspectives of stakeholders, including traditional and alternative/complementary providers, who provide a wide range of supportive and clinical services to racially and ethnically diverse pregnant people in Alabama across the continuum of maternal care.

## Methods

### Overview

This study's methods and results are organized according to the Consolidated Criteria for Reporting Qualitative Research (COREQ) checklist.^17^ This study was approved by the University of Alabama at Birmingham's Institutional Review Board (IRB-3006402).

### Research team positionality and reflexivity

Two interviewers with previous experience conducting qualitative research were trained in interview techniques by qualitative expert investigators. One is a doctoral student (MD, MPH) who identifies as black, while the other is a health disparities researcher (PhD, MPH) who identifies as white. Both identify as cisgender women and shared their interest in reducing maternal health inequities with participants. The researchers acknowledge the possibility that their own racial backgrounds could have influenced the interpretation of the data and made efforts to disclose and discuss their potential biases. They piloted the interview guide, took notes during interviews, drafted postinterview memos, and held regular team discussions throughout piloting, data collection, and analysis to reflect on emerging themes and adjust the guide and interpretations as needed.

### Study design and theoretical framework

Howell's conceptual framework underpins the study and was utilized to develop the semistructured interview guide (Supplementary Data S1).^16,18^ Topics included perspectives on why disparities in SMM persist, contributing multilevel factors, and strategies to overcome the barriers to reduce SMM in Alabama.

### Participant selection

Participants were purposively selected as relevant interested parties through recommendations by study investigators who were not directly involved with conducting or analyzing the interviews. In some cases, participants had a previous professional relationship with one or more investigators (e.g., membership in maternal health workgroups). Eligible participants were (1) those who provided clinical maternity care or nonclinical maternal health services (i.e., lactation counseling, doula services), (2) representatives of maternal health roundtable and review panels, and (3) staff of community-based organizations and government agencies. Recruitment was not restricted by race, ethnicity, gender, or institutional affiliation. Study participants were also asked to recommend additional service providers for consideration.

The interviewers called or emailed potential participants (*n*=37) as part of recruitment but had no other previous relationship with them. Seventeen individuals did not respond or were unavailable to participate. Participants were informed about the purpose of the study during recruitment and provided informed consent at the start of the interview.

### Setting and data collection

The interviewers conducted pilot interviews with two volunteers from relevant fields (an obstetrician gynecologist [OBGYN] and a doula from a community-based organization). Both pilot interviewees identified as black, had experience with interviews, and provided feedback on interviewing skills and the content in the guide.

Participants completed a brief oral demographic survey and an in-depth interview lasting up to 90 min via Zoom at their homes or workplace. Participants were compensated with a $20 gift card. The survey asked for relevant demographic characteristics, including educational experience, current professional role, years of experience, workplace setting, and type of maternity services provided. They were also asked how they identified regarding gender, race, and ethnicity. No notetaker was present, but the interviewer took informal field notes and drafted postinterview memos to aid the team discussions. Study personnel frequently met to discuss main themes, areas to follow-up in future interviews, and to determine saturation, defined as no new themes emerging (*n*=20 interviews).

### Data analysis

Audio files were transcribed using an automated transcription option or a professional transcription company. The interviewers/analysts utilized the postinterview memos, themes from the transcripts, and the interview guide to develop the initial coding framework. Themes from transcripts related to participants' perceptions were organized by level following the overall study conceptual framework.^16,18^ Major themes were identified as broad codes and were refined into fine codes. Using NVivo 12, study team members double-coded six transcripts.^19^ Areas of disagreement were discussed with both coders and a third member of the study team before a revised coding framework was agreed upon to address issues of inter-rater reliability. The remaining transcripts were coded independently, with study team discussions to clarify coding and interpretation as needed. As new fine codes were identified, all transcripts were revisited and coded using the complete coding guide.

The final themes and subthemes relevant to community and systems factors are described in the results, with findings at provider and health care organization levels presented elsewhere.^20^ Participants were not asked to provide feedback on the study findings.

## Results

### Participant characteristics

All enrolled participants completed the interview (*n*=20) from January to March 2021 (Table 1).^20^ Participants identified as obstetric providers, community-based organization staff, or others (public health, researcher, etc.). Most participants identified as female, held graduate-level or professional degrees, and primarily worked in urban or mixed urban and rural settings serving diverse populations across the state. Most reported providing clinical and/or supportive maternity care services with services stretching across the continuum of care as defined as pregnancy, delivery, and postpartum. Some participants reported active membership in local maternal health workgroups.

**Table 1.** Characteristics of Study Participants

Characteristics	***N***	%
No. of participants		
Total	20	100
Gender		
Female	18	90
Male	2	10
Age category		
20–29	2	10
30–39	5	25
40–49	7	35
50–59	4	20
60–69	1	5
70–79	1	5
Birthplace		
Alabama	11	55
Other U.S. states	5	25
Non-U.S. states	4	20
Highest level of education completed		
Associate degree	2	10
Undergraduate	4	20
Completed graduate or professional	14	70
Race		
Black or African American	9	45
White	9	45
Other (self-identified as African and Hispanic/Latino)	2	10
Ethnicity		
Non-Hispanic	19	95
Hispanic	1	5
Maternal health services provider type^a^		
Clinical provider^b^	12	60
Alternative and/or complementary provider^c^	7	35
Medical case worker	1	5
Nonprovider^d^	3	15
Administrative role^e^		
Yes	12	60
No	8	40
Length of time on current job^f^		
1–5 years	12	60
6–10 years	5	25
>10 years	6	30
Workplace location		
Urban	14	70
Rural	0	0
Mixed urban and rural	6	30
Population served by insurance type		
Medicaid	12	60
Other: private insurance, unspecified or not applicable^g^	8	40
Population served by race^h^		
Black or African American	10	50
White	1	5
Hispanic or Latino	6	30
Asian American	2	10
Mixed (across different races and ethnicities)	15	75

The demographic characteristics in Table 1 are included in part from Toluhi et al. (in press), a complementary paper.

^a^ Many participants held multiple roles.

^b^ Physician, nurse, nurse practitioner.

^c^ Doula, certified lactation counselor, birth educator.

^d^ Public health agency staff, not-for-profit representative.

^e^ Director, co-/founder, educator, researcher, public health agency staff.

^f^ Some participants have dual roles with different lengths of time on their current roles.

^g^ Doula services are not covered by Medicaid in Alabama. Clients paid out-of-pocket or their services were covered by grants according to participants.

^h^ Some participants shared the race of their patients/clients when asked about the population they served.

Self-reported race was collected due to the focus on racial disparities to put comments in the context of the participant's race. Race and ethnicity were based on responses to the open-ended questions about how the participants identified their race and ethnicity. Due to the small sample size, individuals who did not fit into the two main groups, black and white, were grouped into the “other” category (Table 1).^20^

### Themes around community and systems factors contributing to racial disparities in maternal mortality

Racism, unjust laws and policies, and poverty and lack of community infrastructure emerged as themes at the community and systems levels as contributing to disparities in maternal health. Participants also suggested strategies at these levels to reduce inequities. Illustrative quotations for major themes and select subthemes are presented in Tables 2 and 3. Many sub/themes overlapped.

**Table 2.** Factors Contributing to Inequities in Maternal Mortality and Severe Maternal Morbidities in AL

	Theme	Brief descriptor	Key representative quote
1	Racism	Insurance is part of institutionalized racism	“Insurance, which again from an institutionalized racism thing, comes through employer, which comes through your availability to either become qualified for a job or to pursue education that makes you qualified for that job, which relates back to the quality of your education, and your opportunities, and what your expected role is, and taking care of your family—the availability of your partner to allow you to go work.” (OBGYN, white)
2		Racism in modern policymaking	“It's a moral majority issue of legislators thinking that if you provide health care to people they're going to stop. They're going to be lazy and stop working. …That's the policy culture of if we provide these things, then the people that we don't think deserve X, Y and Z are going to use the system for their benefit, which we know is not the case. We know that people that have health care coverage are more productive…The data is there. We just have policymakers that don't believe it … It's really rooted in just bigotry and hatred” (Clinical Administrator, white).
3		Cultural disempowerment of voices who are black	“Oftentimes our White counterparts have been given the right to express what they want. They have [been] given the right to and they had privileges to feel like you should hear. When oftentimes African American women don't feel like we have that right. So that's an internal statement I think plays a huge part in why a lot of infant mortality exists individually as far as our community [is concerned]” (Doula/Researcher, black).
4		Policies impacting maternal health were created without diverse interested parties involved	“Many of our policies were routed back in the Jim Crow Era. Most of our policies did not have us in mind. So when you are dealing with individuals or institutions who have fundamental background and fundamental foundations that did not have us in mind, then we're not a part of it, and not a part of it will not allow for our concerns to be addressed” (Doula/Researcher, black).
5	Unjust laws and policies	Lack of diverse representation, including provider type, in change organizations	“When you have [Maternal Mortality Review Board] packed with physicians only and when you ask why are non-clinicians not a part of that group—whether that's families or doulas or other birth support people that have insight that will give you insight into the level of the community and the relationships and the and the individual factors that might contribute to someone you know being listed or their cases being reviewed. There's not a lot of support for Non clinicians to be a part of those groups” (Doula/CBO Administrator, black).
6		Lack of Medicaid specialty and postpartum coverage (subtheme: health insurance policies)	“A woman who had peri cardiomyopathy and congestive heart failure and she should have been receiving lifetime cardiac care, but when her insurance lapsed at six weeks, all of a sudden, she couldn't. …She wound up having a heart attack and passing away. That's a policy result because she was unable to access the care that she needed postpartum, [supporting the need for] expansion of Medicaid.” (Clinical Administrator, white).
7		Policies restrict care (subtheme: health insurance policies)	“The ability to be able to choose a doctor that aligns with and would support more physiological birth was curtailed by the state because they said you can't cross county lines and made other restrictions like you could only change doctors, a certain or a limited amount of time. Patients were really cautious about that. Usually, they had chosen their doctor before choosing a doula and it can take some time to petition to choose a different doctor…If they want to leave one doctor and go to another they might miss several prenatal appointments, which we know, especially with low uptake of early prenatal care can be detrimental to women of color and [increase] fetal or maternal mortality” (Doula/CBO Administrator, black).
8		Policies restrict services (subtheme: other restrictive laws and policies)	“I think that there is a massive role, for issues associated with our abortion laws. I think that does play a huge role because you see a lot of cases where honestly a mom should have been counseled to maybe not continue a pregnancy because she was in such severe cardiac failure or had severe autoimmne disease or something. We can't have those conversations…How do we actually advocate for the health of moms when you can't give them all the information?” (Clinical Administrator, white).
9		Inflexible policies restrict voices, services, and lead to unintended pregnancy (subtheme: other restrictive laws)	“there's a policy within Medicaid that you have to wait a certain time, even if you want tubal ligation,…which is something that a woman is saying that she wants, she does not want more children. That's something that if you end up having more children, it could contribute to—maybe if let's say like you're struggling really economically and you feel like I just can't afford another kid and you end up having one, that can have an impact in your economic future or many other things. That's something that we've seen that women are saying that they want that, but then there's this policy barrier there that they're not able to get it and they end up pregnant again. Then you continue the cycle of unintended pregnancy, so it can—and sometimes women have chronic conditions and it's something that can either this can be aggravated or their conditions can aggravate their pregnancy. It's just like this cycle. There are reasons why that specific policy was put in place, but it's like an example of this inflexibility or rigidity in the system, that's not really accounting for women's desires and voices. I think it was put there because of coercion in the past, but then it's still not working for people and it can end up putting them in a situation that they're trying to avoid.” (NFP Administrator, other).
10		Lack of parental leave policies (subtheme: other restrictive laws and policies)	“Maternity leave is just so little here in the United States. When I was practicing in California, you could get disability six weeks paid disability for maternity leave. But in Alabama we don't get that. Our patients don't get that. So I think FMLA is great, but it's unpaid and most of our patients can't take unpaid time off. I think that's a huge thing barrier—they don't go to their appointments postpartum. They don't get the birth control. Then they get pregnant again and then….” (OBGYN, white).
11		Chemical endangerment laws criminalize pregnant people (subtheme: other restrictive laws and policies)	“Every county has different policies, if you're looking at things like arrests for substance use, based off of our chemical endangerment laws…. Alabama has child chemical endangerment laws, which were really put in place years ago in order to prevent kids that were killed or injured if you have meth lab…I mean that is something that does absolutely make sense, … Alabama has interpreted it to fetuses saying that if you are using substances, while pregnant, you are endangering the life of a child, and you deserve that same punitive punishment. So, again it's a big idea of our lobbyist actually say that if it doesn't deal with guns or gynecology, our government oversight Alabama doesn't really care. And it's true. Legislators right now that are trying to limit the power of the health directors at the state and county level….” (Clinical Administrator, white).
12		Policies impact provider, care (subtheme: other restrictive laws and policies)	“I hear all the time that most physicians are getting underpaid in low-income communities, because most of them are Medicaid [and have poor reimbursement policies]. So they're overworked and underpaid…It's not that all of the physicians don't care about their need. It is just that they have so many patients to care for that they don't know how to give to all” (CBO Administrator, black).
13	Poverty and lack of infrastructure	A lack of safe place to be active and access nutritious foods leads to overall poor health	“The neighborhoods are more likely to not have parks, to not be safe to be outside, to not have supermarkets walking distance away. I mean you just need to drive around Birmingham and look at the difference between Mountain Brook and Ensley. Then you tell me why it's more stressful to live in Ensley than in Mountain Brook…Obesity is a huge problem. But that's an access to healthy food problem” (CBO Administrator, white).
14		Lack of transportation	“I can think of one particular patient who was pregnant with triplets that had a two year old at home. She had no help whatsoever. She didn't have transportation. We would arrange transportation for her and she just…Education was lacking hugely. She didn't take the triplets back for their follow up appointments and by the time they came to see us for her postpartum appointment, we ended up admitting them to the hospital [for malnourishment],” (Nurse, black).

CBO, Community-Based Organization.

**Table 3.** Participant-Proposed Strategies to Reduce Inequities in Maternal Mortality and Severe Maternal Morbidities in Alabama

	Theme	Brief descriptor	Key representative quote
1	Incorporate care into communities	Explore an alternative model of care.	“In the UK, they have their—I think their model of maternity care is very midwife heavy and they've made great progress in reducing their maternal mortality. I think then other places have used community health workers a lot and that's not someone that's gonna ever replace a physician, but there's a big role they can place anyways in education and empowerment, connecting to services, different things like that” (Program Director, Other).
2		Explore an alternative model of care.	“But in reality, what you should have is a pyramid system where you have your low risk births are attended by nurse midwives. It saves money. It improves outcomes. Patient satisfaction goes up all because [of] that midwifery model. If you have slightly higher risk patients or patients that really just want to see an OB great here's your middle tier…then you have your MFS [maternal fetal medicine specialists] up at the top, where you they're seeing the most high risk patients….and then there's a place for home births, there is a place for Certified midwives and certified professional midwives…” (Clinical Administrator, white)
3		Address the needs of rural areas to bridge gaps in care.	“The majority of [Certified Nurse Midwives] are in the Jefferson County. We have plenty of obstetrical services in Jefferson County. They need to be out in the rural communities”(OBGYN, black).
4		Address facilities in rural areas.	“We have now a clinic in [rural county]. And that is new, as of the past year, and so a lot of those patients were having to travel far distances to get their OB care” (OBGYN, white).
5		Explore an alternative model of care.	“Making some type of program that's with the hospital. They maybe have transportation like a bus or some type of vehicle where they go or do home visits, they have the option to go do home visits” (CBO Administrator, Certified Lactation Counselor, black).
6		Utilize trusted sources of information to meet people where they are.	“But what we need [is] our navigators and the community. We need…cultural brokers people who can actually get into these places—whether it be churches, community centers. I think we've got to do this real grassroots type of effort. In these areas, where you meet people where they are” (OBGYN, black).
7	Emphasize social services	Learn from global lessons to support families through infrastructure	“Mom has to be okay, because if she's not okay in the family, then the family kind of all falls apart… Like what's the phrase that we use here? It's kind of family values, …Of course, the Netherlands is not the US and they've got all the infrastructure and what have you. But, these again are things that that we can learn, and but they've got these Community Centers where they can drop the children off and they're cared for and give mom some time with the newborn…We can certainly study those things and try to implement them here in our country. I think we can do a better job just caring for families, I think, as a whole.” (OBGYN, black).
8		Social services should continue to address mental health, substance abuse disorder, and nutrition insecurity.	“Yeah. If available services include social services? I think so. There's so many different needs. It could be things that can support you if you're pregnant or have a baby, like WIC, for example, or having home visiting in your community, or having different things like that. Folks that can provide different services. If those support services are not there or if you're somebody that has a substance use disorder or mental health, you need counseling or something like that. Something like mental health that can affect your physical health as well and so if you don't have that in your community, either there's nobody there, or there isn't somebody that you trust, then yeah, that can affect your health. You may not be able to—for the case of mental health, then it can prevent you from being in a place where you can access other services or—” (NFP Administrator, other).
9		Reduce financial burdens.	“People who better financially are able to eat or they're able to afford transportation, so that in an emergency a medical emergency or a prenatal emergency that they're able to go to the doctor when they need to. They can make their health appointment” (Doula/CBO Administrator, black).
10	Expand health coverage	Expand Medicaid to cover postpartum period and specialty care.	“Medicaid expansion—…It is helpful because if I need her to get a CT scan, for example, or if I need to get her referred for a procedure, she still has coverage that goes with that, and…. They're not getting a bill for thousands of dollars. They're getting the copay that they have to pay. So funding its expansion, where doable and again that's a thing many people above me and public policy are talking about and trying to think through. ‘How do you make it fiscally responsible, but also generous to people that need it to not leave people on the cold’. But I think practically speaking when people have Medicaid insurance it's easier to do things like can get contraception easier, which is a big thing from a maternal mortality morbidity perspective” (OBGYN Resident, white).
11	Enact equity-centered policies to support health and reduce racial disparities	Parental leave is evidence-based and should be standard.	“What we know, is it from looking at other countries that are doing better around maternal mortality is the addition of parental leave as a standard so whether that's nationally or within a state and several states have stepped up to provide that to families. So that moms don't have to go back to work within three days of giving birth, which definitely increases their risk of for a lot of different things” (Doula/CBO Administrator, black).
12		Remove laws that criminalize pregnant women.	“I understand the state screening is not being done universally…I don't know that they're using validated tools, which is what's recommended by ACOG and ASAM. I think that you use—you do universal screening, not drug testing, and you use validated tools. The state is it's difficult though because we have these laws that criminalized drug use for pregnant women.…I know that there there's folks working at the policy level to remove those laws, and hopefully, that happens.” (NFP Administrator, other).

ACOG, American College of Obstetricians and Gynecologists; ASAM, American Society of Addiction Medicine; FMLA, Family and Medical Leave Act; NFP, not-for-profit; OBGYN, obstetrician gynecologist; WIC, Women, Infants, & Children.

## Racism

Systemic racism present in Alabama was discussed as a driving factor behind racial disparities in maternal health by many of the service providers. Some participants described the health insurance system as a part of institutionalized racism (Table 2, Quote 1). They noted that the legacy of racism included the lack of racial diversity in many communities, issues of environmental justice that contribute to overall poor health of people who are black, cultural disempowerment of black voices (Table 2, Quote 2), the racism of policymakers and lack of diverse interested parties involved in policymaking (Table 2, Quote 3–4), as well as gerrymandering as preventing active participation of communities of color in policy change.

## Unjust Laws and Policies

Participants described several laws and policies as unjust and contributing to racial disparities in maternal health in Alabama. Many identified specific laws and policies (i.e., health insurance and paid parental leave) as contributing to the issue. Some providers suggested that these unjust laws and policies were related to the lack of inclusion of diverse participants in influencing organizations such as the maternal mortality review programs (Table 2, Quote 5).

### Health insurance policies

Many participants spoke about their experiences working with black pregnant/postpartum individuals as well as their experiences with people with inadequate health coverage. They noted that in Alabama harmful policies included lack of insurance coverage during the postpartum period, restrictions on choice for provider, restrictions on changes to provider or facility, lack of coverage for complementary and alternative services, lack of insurance for undocumented people, and distance to providers covered by health insurance as contributing factors (Table 2, Quotes 6–7).

### Other restrictive laws and policies

Participants noted laws restrict women's access to abortion in Alabama, with some unable to travel across state lines for services (Table 2, Quote 8). These inflexible policies limit pregnant individuals' autonomy and contribute to undesired pregnancies (Table 2, Quote 9). Participants also described using chemical endangerment laws to criminalize pregnant people (Table 2, Quote 10), as well as prosecuting pregnant patients with substance use disorder and sending them to jail without rehabilitation services or medicated assisted therapy as unjust. They discussed the lack of standardized paid parental leave as putting the health of pregnant individuals at increased risk when they return to work mere days after delivery to meet their families' financial needs (Table 2, Quote 11).

Some participants noted that Medicaid reimbursement policies led to doctors being underpaid and overworked (Table 2, Quote 12). In sum, participants shared their belief that these policies disproportionately affect low-income black women in the Deep South.

## Poverty and Lack of Infrastructure in Black Communities

Participants discussed poverty and resource insecurity (housing, nutrition, transportation, etc.), which disproportionately impact certain communities in Alabama. They explained that these issues might lead to the inability to comply with provider recommendations and interfere with the continuity of care, which sometimes results in negative judgments, stigma from members of the health care community, delays in care, and an increased need for emergency services. They noted issues around the lack of safe environments in some communities due to crime and lack of sidewalks and lights. They felt these contribute to stressful environments, mental health concerns, and the inability to be active.

Some service providers elaborated that the lack of grocery stores creates food deserts and contributes to obesity and comorbidities, which can contribute to poor maternal health (Table 2, Quote 13). Participants also noted that these issues are exacerbated by the lack of transportation or medical facilities within neighborhoods of lower income (Table 2, Quote 14).

## Suggested Strategies to Overcome Community- and Systems-Level Barriers

### Incorporate care into communities

Participants emphasized strategies to increase access of black pregnant individuals to maternity care. They suggested including members of black communities in the provision of maternity care, which would require systems-level interventions. Some advocated for a pyramid model of care, which would include community health workers, a care team including complementary and alternative providers (certified nurse midwives, doulas) to support low-risk births, and OBGYNs and maternal–fetal medicine specialists to see high-risk patients (Table 3, Quotes 1–2). Others mentioned the need to advocate for a stronger role for nurse practitioners. To reach rural areas or areas lacking specialty facilities, participants suggested telemedicine and changing laws to support the use of certified nurse midwives (Table 3, Quote 3–4).

Participants noted the importance of bringing care to where pregnant individuals live. This can be done by locating health facilities within communities and preventing hospital closures, giving the option for home visits, and improving transportation with options such as group transportation shown to be effective in other settings (Table 3, Quote 5). Other suggestions included resourced medical buses, transport provided by hospital networks, and improvements in the timeliness, reliability, and pregnant individuals' awareness of existing transportation services. They noted that working with trusted sources of information within communities, both informal and formal (i.e., faith-based leaders), would be essential to increase dialogue between communities and medical care providers and systems. Engagement should establish specific needs and ensure that strategies, resources, and communication are culturally appropriate and relevant (Table 3, Quote 6).

### Emphasize social services

Participants noted that social services should be emphasized during prenatal care to support the whole family with childcare, health coverage, and programs impacting one's overall health (i.e., mental health, substance use disorders, nutrition, and housing insecurity) as those can affect one's ability to utilize maternal health services (Table 2, Quotes 7–9).

### Expand health insurance coverage

Participants all agreed about the need for Medicaid expansion in Alabama and similar states. Participants recommended extending coverage beyond 6 weeks during the postpartum period, allowing for free use of specialty services, and providing coverage of services by nontraditional providers in low-risk and medically underserved settings (Table 3, Quote 10).

### Enact equity-centered policies to support health and reduce racial disparities

Participants advocated for antiracism in health policy as a critical strategy to reduce egregious disparities. They called for more diverse voices in policymaking at all levels. Participants noted the need to standardize paid parental leave policies (Table 3, Quote 11), remove policies that criminalize pregnant people (Table 3, Quote 12), and remove policies that restrict provider and facility choice.

## Discussion

Participants provided nuanced, descriptive data on community and systems contributors to the persistent inequity in maternal health in Alabama and other Deep South states. Specifically, this study identifies the heightened impact of racism in the Deep South context and how it is reflected in systems, laws, policies, and communities. These findings highlight the complexity of the issues and point to potential strategies to reduce them. All emphasized the need for approaches to be evidence-based and modeled from other states and countries. Our study participants identified the issue of structural racism to be a driving force in inequities in maternal health in Alabama, which has been documented in other parts of the country.^21–25^ However, systemic racism is particularly salient in the Deep South, with inadequate desegregation leading to reduced care access and quality.^26^

More recently, many states in the U.S. South did not expand Medicaid, which according to Nolen et al. accounts for 92% of adults in the coverage gap; these states are also disproportionately impacted due to the more significant number of black people, with black populations more likely to be underinsured compared with other populations.^27–29^ The concerns around systemic racism and neighborhood-level segregation align with evidence of SMM being associated with racialized economic segregation, which requires policy initiatives to overcome.^30,31^

The consensus for care in communities dominated many stakeholder interviews and aligns with existing literature advocating for equity-centered approaches to care.^32,33^ The community-informed care models described by participants, which provide team-based care and have home visiting options, support human rights from the reproductive justice perspective.^34^ In-home visiting programs with community health workers or nurses have effectively improved prenatal care utilization and birth outcomes such as preterm birth among economically disadvantaged and ethnically diverse women.^35–37^ The need for policy changes to increase access and affordability of maternity care was highlighted by our participants and has been emphasized by others.^25,38^ Additional strategies to reduce inequities from the literature include prioritizing implementation research and promoting opportunities for providers to have meaningful discussions with patients outside of the clinical setting.^38,39^

The findings of this study should be interpreted considering its limitations. Participants focused discussion on issues within Alabama, which has a unique context related to health access and infrastructure needs.^40^ Participants were purposively recruited through their active participation in professional roles related to maternal health care. Many participants worked with populations of lower income pregnant women in urban or mixed urban and rural settings and described barriers and strategies related to economic disadvantage. Yet, many black families have a high socioeconomic status yet still experience inequities in SMM, which further supports the need for systems-level solutions and strategies to reduce systemic racism.^15,41^

Despite efforts to increase comfort in the discussion of sensitive topics, including racial concordance in half of the interviews, participants may have been hesitant to disclose factors that could be perceived as contributing to the issue themselves, considering the heightened social discord around issues of racism in the United States at the time of interviews.^42,43^ Despite efforts to recruit racially and ethnically diverse participants across genders, the participants primarily identified as women and black or white.^44,45^ Interviews focused on black cis-gender women, but maternal health inequities are possible across the spectrum of gender identities; more research is needed to understand their unique challenges.

Interview data reflect the situation at the time of data collection. Although most remain relevant, encouraging efforts include establishing the Alabama Maternal Health Task Force and Medicaid extension.^14,46^ The extension includes dental coverage for pregnant individuals, postpartum coverage up to 1 year after delivery, and reimbursement rate increases for maternity health care professionals.^47–50^ These experts' calls to expand Medicaid eligibility are promising, but would be insufficient with other systems-level interventions needed to improve pregnancy and birth outcomes and reduce racial disparities.^51–54^ In addition, Alabama residents face greater challenges to reproductive rights, including further restriction on abortion access in the state enforcing a total abortion ban with anticipated consequences, including increased maternal and infant mortality and reduced access to early and continuous prenatal care and birthing services.^55–57^

### Health equity implications

These stakeholder perspectives provided contextual, community, and systemic factors often unavailable in other maternal health surveillance systems. Contributors identified include systemic racism, barriers to access to care such as a lack of transportation and insurance coverage, and policies restricting pregnant individuals' choices. Our informants described actionable strategies and emphasized that these should be evidence based, community focused, and culturally appropriate. Strategies should focus on improving pregnant individuals' overall health across the life span and include broad and multisectoral policies to address issues of systemic racism, expand Medicaid, and incorporate choice for services for pregnant individuals.

Lastly, these locally informed findings can guide discussions with health system and community partners about Alabama and other Deep South local and statewide initiatives to reduce racial disparities in maternal health.

## Supplementary Material

Supplemental data

## Abbreviations Used

ACOG: American College of Obstetricians and Gynecologists
ASAM: American Society of Addiction Medicine
CBO: Community-based organization
COREQ: Consolidated Criteria for Reporting Qualitative Research
FMLA: Family and Medical Leave Act
NFP: not-for-profit organization
OBGYN: obstetrician gynecologist
SMM: severe maternal morbidity and mortality
WIC: Women, Infants, & Children
