# Supplementary material for: Community and Systems Contributors and Strategies to Reduce Racial Inequities in Maternal Health in the Deep South: Provider Perspectives
Source: Health Equity. 2023 Sep 13;7(1):581–91. doi: 10.1089/heq.2023.0114 (PMC10510686; doi:10.1089/heq.2023.0114)
Supplement: Supplemental data [file Suppl_DataS1.docx]

Supplemental 1. Interview Guide & Demographic Questionnaire

**I. INTRODUCTION**

My name is ___________________. I am working with the XXX on a project aiming to understand and address disparities in maternal health in Alabama. We would like to talk to you about racial disparities in maternal health outcomes in our state and why they occur. We would also like to learn about your perspectives on potential interventions and programs that could reduce these disparities. Everything you share during this interview will be kept confidential and will not be shared with your employer or other colleagues afterwards, except in summary form with no names or other identifiable information included. This interview will take up to one and a half hours.

Confirm that the study has an informed consent document on file for this participant.

(Ask permission to audio record the discussion, and if they agree, start the recorder AFTER any introductions. This guide includes the topics to be covered and questions that may be helpful in facilitating the interview. You do NOT have to ask all the questions or follow the order given in the guide. Always elicit spontaneous responses thoroughly before moving to probes.)

**II. INTERVIEW TOPICS**

1. **Maternal Health Role**
   1. Please tell me about the work you do related to maternal health in Alabama.
   2. What populations do you serve?
   3. What do you do in your work related to Pregnancy care? Delivery care? Postpartum?
2. **Racial disparities**
   1. In the course of your work what differences do you see (if any) in maternal and infant outcomes--such as Maternal Mortality and Severe Maternal Morbidity--by race?
   2. What types of adverse maternal health outcomes do you see happening in Black patients?

Data from the ADPH show that Black patients have higher rates of maternal mortality and severe maternal morbidity than White patients in AL. Alabama is currently ranked as the having the third highest maternal mortality rate – after Arkansas and Kentucky [44]. AL has a maternal mortality ratio of 11.9 per 100,000 live births [45]. However, when this is reviewed in terms of race, white patients have a maternal mortality rate of 5.6 while black patients were five times more likely to fire from pregnancy related causes with a MMR of 27.6 per 100,000 LBs. It is important to note that black patients who earn higher incomes still dies at higher rates than white patients of the same economic background [46]. What do you think are some of the major reasons that Black patients have worse maternal health outcomes in our State?

1. **Contributing factors to racial disparities**
   1. Please tell me about any individual patient factors that you think contribute to these racial disparities in maternal health outcomes? Probes:
      - Patients’ socio-demographics? (age, education, income, etc.)
      - Patients’ knowledge and beliefs?
      - Patients’ psychosocial resources (like self-efficacy and social support)?
   2. Please tell me about some aspects of the communities or neighborhoods where Black patients live that might contribute to these racial disparities in maternal health outcomes? Probes:
      - Community-level healthcare trust or distrust?
      - Neighborhood factors (housing, built environment, transportation, stores, etc.)?
      - Available health and social services in the community?
      - Racial diversity in the neighborhood?
   3. Please tell me anything about the way some healthcare providers practice or deliver services that might contribute to might contribute to these racial disparities in maternal health outcomes? Probes?

**Years of experience**? Years since training?

**Cultural (in)competency** (the ability to understand, appreciate and interact with people from **cultures** or belief systems different from one's own)

**Structural (in) competency** (the ability to discern how a host of issues defined clinically as symptoms, attitudes, or diseases also represent the downstream implications of a number of upstream social determinants of health)

**Explicit (conscious) and/or Implicit (unconscious) bias?**

**Intersecting stigmatizing** beliefs, attitudes, and stereotypes related to race, ethnicity, poverty, substance use, mental illness, etc.

Lack of good **communication skills**? Shared decision-making skills?

Familiarity with **trauma-informed care practices**?

Knowledge about **evidence-based maternal health interventions**?

- 1. Please tell me anything about how maternity services are organized at hospitals and other health facilities that might contribute to these racial disparities. Probes:

Provider-patient racial and/or language concordance/discordance (OB provider and RNs)

Diversity of care provider types (midwives v. NPs v. physicians)

Birthplace/setting

Options and collaborative agreements (hospital v birth center v home birth)

Workforce shortages

Visitor policies – especially during COVID

Drug screening policies

Differing care options based on insurance status

Informed consent policies

- 1. What about any bigger systems-level factors (things related to local, state, or federal laws, policies, health and insurance systems, payers, etc.) that might contribute to these disparities? Probes:
     - - - Health insurance? Medicaid coverage?
         - Quality of health care for poor people?
         - Coordination of care?
         - Transportation systems?
         - Policies?
         - Laws?
         - Structural racism?

1. **What Can Be Done?**
   1. Based on your experiences, observations, and insights, what do you think should be / can be done to reduce the racial disparities in maternal health in our state?
   2. Finally I want to get your feedback on some strategies that have been proposed to reduce MM/SMM disparities in AL. For each of these strategies, can you tell me if you think it might be feasible? acceptable? Effective in reducing racial disparities in maternal health outcomes?
   - Bundled safety and quality measures (e.g., early warning sign criteria, simulation training, coordinated care)
   - Cultural and/or structural competency training for providers
   - Implicit bias training for healthcare providers
   - Implementing a disparities dashboard
   - Tailored home visiting programs for pregnant and postpartum patients
   - Electronic health solutions (apps for providers and/or patients)
   - Other strategies?

**III. CLOSING**

Thank you very much for your time. Your responses will be very helpful for improving the health of families in Alabama. Now, I’ll turn the recorder off and ask a few demographic questions about you. (Turn off recorder and complete the participant characteristics).

**Participant Characteristics**

| Study ID# |  |
| --- | --- |
| Type of participant  (circle all that apply) | 1. UAB obstetric care provider 2. Non-UAB obstetric care provider 3. Jefferson County MCH Roundtable member 4. Member of AL Maternal Mortality Review Program 5. Community-based organization representative 6. Other: (please describe) |
| Gender | 1. Male 2. Female 3. Transgender (please describe): 4. Other (please describe): |
| Age |  |
| Birthplace |  |
| Highest level of education completed | 1. Did not complete high school 2. Completed High School or GED 3. Completed Some College Courses or an Associated Degree 4. Completed Undergraduate Degree 5. Completed Graduate or Professional Degree |
| Terminal Degree |  |
| Workplace | 1. Urban area 2. Rural area 3. Mixed urban and rural |
| Race | 1. American Indian or Alaska Native 2. Asian 3. Black or African American 4. Hispanic or Latino 5. Native Hawaiian or Other Pacific Islander 6. White |
| Ethnicity | 1. Hispanic 2. Non-Hispanic |
| Main occupation / position |  |
| Length of time in current job |  |
| Type of maternity services provided | 1. Pregnancy care 2. Delivery care 3. Postpartum |
